# Accuracy of FAST scan in blunt abdominal trauma in a major London trauma centre

**DOI:** 10.1186/cc12228

**Published:** 2013-03-19

**Authors:** S Fleming, R Bird, K Ratnasingham, S Sarker, M Walsh, B Patel

**Affiliations:** 1 Royal National Orthopaedic Hospital Stanmore, London, UK; 2 Royal London Hospital, London, UK; 3 Barts Cancer Institute, London, UK

## Introduction

Blunt abdominal trauma (BAT) is a leading cause of morbidity and mortality. Rapid diagnosis and treatment with the Advanced Trauma Life Support guidelines are vital, leading to the development of focused assessment with sonography in trauma (FAST).

## Methods

A retrospective study carried out from January 2007 to 2008 on all patients who presented with BAT and underwent FAST scan. All patients subsequently had a CT scan within 2 hours of admission or a laparotomy within 2 days. The presence of intraperitoneal free fluid was interpreted as positive.

## Results

One hundred patients with BAT presented; 71 had complete data. The accuracy of FAST in BAT was 59.2%; in these, 31 (43.7%) were confirmed by CT and 11 (15%) by laparotomy. There were 29 (40.8%) inaccurate FAST scans, all confirmed by CT. FAST had a specificity of 94.7% (95% CI: 0.75 to 0.99) and sensitivity of 46.2% (95% CI: 0.33 to 0.60). A positive predictive value of 0.96 (0.81 to 0.99) and negative predictive value of 0.39 (0.26 to 0.54). Fisher's exact test shows positive FAST is significantly associated with intra-abdominal pathology (*P *= 0.001). Cohen's chance corrected agreement was 0.3. Twenty-one out of 28 who underwent laparotomies had positive FAST results, indicating accuracy of 75% (95% CI: 57 to 87%).

## Conclusion

Patients with false negative scans requiring therapeutic laparotomy is concerning. In unstable patients, FAST may help in triaging and identifying those requiring laparotomy. Negative FAST scans do not exclude abdominal injury. Further randomised control trials are recommended if the role of FAST is to be better understood.
